# The Impact of Bedbug (*Cimex* spp.) Bites on Self-Rated Health and Average Hours of Sleep per Day: A Cross-Sectional Study among Hong Kong Bedbug Victims

**DOI:** 10.3390/insects12111027

**Published:** 2021-11-14

**Authors:** Eddy Hin Chung Fung, Siu Wai Chiu, Hon-Ming Lam, Roger Yat-Nork Chung, Samuel Yeung Shan Wong, Siu Ming Chan, Dong Dong, Hung Wong

**Affiliations:** 1 JC School of Public Health and Primary Care, The Chinese University of Hong Kong, Hong Kong, China; rychung@cuhk.edu.hk (R.Y.-N.C.); yeungshanwong@cuhk.edu.hk (S.Y.S.W.); dongdong@cuhk.edu.hk (D.D.); 2 Faculty of Law, The Chinese University of Hong Kong, Hong Kong, China; 3 School of Life Science, The Chinese University of Hong Kong, Hong Kong, China; swchiucuhk@gmail.com (S.W.C.); honming@cuhk.edu.hk (H.-M.L.); 4 Hong Kong Poison Information Centre, Hospital Authority, Hong Kong, China; 5 CUHK Institute of Health Equity, The Chinese University of Hong Kong, Hong Kong, China; hwong@swk.cuhk.edu.hk; 6 Department of Social and Behavioural Sciences (Social Work), City University of Hong Kong, Hong Kong, China; chansiuming0609@gmail.com; 7 Department of Social Work, The Chinese University of Hong Kong, Hong Kong, China

**Keywords:** bed bugs, *Cimex* spp., Hong Kong, sleep disturbance, health impact, public health, causal agent, infectious agent, vector

## Abstract

**Simple Summary:**

Bedbugs (*Cimex* spp.) are a nuisance pest of significant public health importance that is on the rise globally, especially in crowded cities such as Hong Kong. Bedbug infestations disproportionately affect underprivileged communities living in crowded and dilapidated housing. This study uses an online survey to investigate the health impacts of bedbug infestations among bedbug victims. This study found that most bedbug victims experienced ≥five bites in the past month, usually on the arms and legs. The most common reaction to bites were itchiness, redness, and swelling of the skin, and difficulties sleeping or restlessness. Bites usually occurred during sleep, impacting the bedbug victim’s mental and emotional health, and sleeping quality most severely. The adverse health outcomes of bedbug infestations were associated with the lower self-rated health and average hours of sleep per day of bedbug victims. This study brings attention to the neglected issue of bedbug infestations by providing evidence on the scope of its health impacts, informing public health interventions including public education and extermination programmes, and supportive laws and policies for adequate housing and hygiene. The successful control of bedbugs in an international city such as Hong Kong can inform the control of the global bedbug resurgence.

**Abstract:**

Bedbugs (*Cimex* spp.) are a nuisance public-health pest that is on the rise globally, particularly in crowded cities such as Hong Kong. To investigate the health impacts of bedbug infestations among bedbug victims, online surveys were distributed in Hong Kong between June 2019 to July 2020. Data on sociodemographics, self-rated health, average hours of sleep per day, and details of bedbug infestation were collected. Bivariate and multivariable analysis were performed using logistic regression. The survey identified 422 bedbug victims; among them, 223 (52.9%) experienced ≥five bites in the past month; most bites occurred on the arms (*n* = 202, 47.8%) and legs (*n* = 215, 51%), and the most common reaction to bites were itchiness (*n* = 322, 76.3%), redness, and swelling of the skin (*n* = 246, 58.1%), and difficulties sleeping or restlessness (*n* = 125, 29.6%). Bites usually occurred during sleep (*n* = 230, 54.5%). For impact on daily life in the past month, most bedbug victims reported moderate to severe impact on mental and emotional health (*n* = 223, 52.8%) and sleeping quality (*n* = 239, 56.6%). Lower self-rated health (aOR < 1) was independently associated with impact on physical appearance (*p* = 0.008), spending money on medication or doctor consultation (*p* = 0.04), number of bites in the past month (*p* = 0.023), and irregular time of bites (*p* = 0.003). Lower average hours of sleep per day (aOR < 1) was independently associated with impact on mental and emotional health (*p* = 0.016). This study brings attention to the neglected issue of bedbug infestation by considering bedbugs as an infectious agent instead of a vector and providing empirical evidence describing its health impacts.

## 1. Introduction

Bedbugs (*Cimex* spp.) are hematophagous ectoparasites that pose a significant threat to public health [1]. The burden from bedbug infestations is expected to worsen due to the global bedbug resurgence which has been attributed to several factors including human population growth, increase in international travel and trade, and urbanisation [2,3,4].

**Figure 1:**
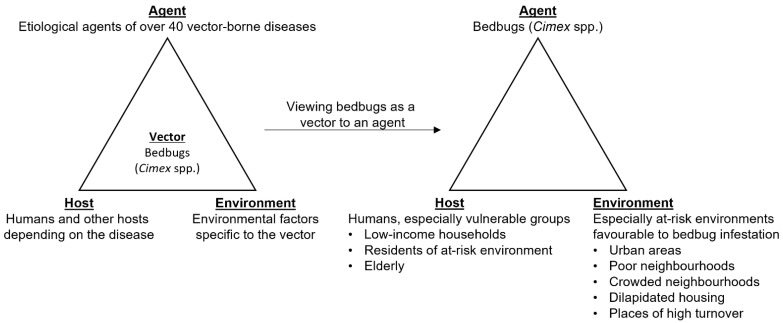
Shifting the view from bedbugs as a vector to an agent.

Although bedbugs have the potential to transmit etiological agents of over 40 vector-borne diseases, no reported outbreaks have been attributed to them [4,5,6,7]. However, the impact of bedbug infestation cannot only be measured by its potential for transmitting vector-borne diseases but as an agent itself (Figure 1).

Previous studies have linked bedbug infestations to adverse health outcomes, including cimicosis—itchy sores that result from bedbug bites—and insomnia, and the economic burden of their extermination which may include hiring exterminators, purchasing insecticides, or replacing infested furniture [1,8,9,10]. A previous cross-sectional study found that the health impact and financial burden of bedbugs is prevalent among its victims where over 70% had experienced health, psychological, social life, and financial impact [11].

Several mental health conditions were associated with bedbug infestations which included general psychological symptoms such as distress or anxiety, and diagnosable psychiatric disorders including post-traumatic stress disorder (PTSD), phobia, and depression [12,13,14]. The financial burden of bedbug infestations can contribute to the development or worsening of these mental health conditions [12].

The health impact of bedbug infestations disproportionately affects low-income households due to environmental and socioeconomic vulnerabilities, such as living in crowded housing, having low income, or low education level [10,15,16,17,18].

Pest-control companies in Hong Kong have reported increased cases of bedbug infestations in recent years [19]. Existing volunteer bedbug extermination services in Hong Kong are limited by human and material resources [20]. The crowded and dilapidated features of many housing buildings in Hong Kong facilitate the spread of bedbug infestations [21]. Bedbug victims, particularly those living in subdivided units, may sleep in 24 h restaurants to escape bedbug bites, contributing to the ‘Mc-Refugee’ phenomena [22,23]. Nevertheless, bedbug infestations have been a neglected issue in Hong Kong [19,23].

There are a lack of data on the patterns of bedbug bites (e.g., location, time, physical reaction) and the health impact they have on bedbug victims in Hong Kong. To bring attention to the neglected bedbug issue and inform public health laws, policies, and initiatives on alleviating bedbug infestation, this study aims to describe the patterns of bedbug bites and their effect on self-rated health and average hours of sleep per day.

## 2. Materials and Methods

### 2.1. Data Collection and Sampling

This study was part of a larger project entitled ‘Providing low-income residents with safe, effective, affordable and sustainable solutions in tackling bed bug problems’ conducted by the Chinese University of Hong Kong (CUHK) Anti-Bedbug Research Action Group. Details of the data collection and sampling methods were reported previously [21]. In brief, this was a cross-sectional study conducted from June 2019 to July 2020 in the Hong Kong Special Administrative Region, China. Online self-reported questionnaires in Chinese were distributed via an electronic link on social media platforms. The back translated English version of the questionnaire used is provided as a Supplementary Material (Supplementary Table S1). The questionnaire collected data on the participants’ sociodemographic, self-rated health, average hours of sleep per day, and history of bedbug infestation including its severity, impact on daily life, and details of bedbug bites. Participants were eligible to participate if they lived in Hong Kong and were aged 18 or above. Participants were recruited by volunteer sampling. After accessing the link to the online survey, participants were shown a consent form which explained the details of the study. Participants provided their informed consent to participate in digital form. A total of 696 participants completed the survey.

### 2.2. Measures

All data were collected as categorical variables. All participants were asked to rate their health status on a 10-point Likert scale with a higher score representing better health, report their average hours of sleep per day in the past month, and how often participants saw bedbugs in their place of residence in the past year with responses ranging from ‘never’ to ‘very often’ on a five-point Likert scale. If participants had seen bedbugs in their place of residence in the past year (i.e., not ‘never’), data would be collected on the severity of the bedbug infestation in the past year, severity of impact on daily life in the past month with regards to some aspects of health including physical health, and mental and emotional health, frequency of bedbug bites, location of bites, reaction to bites, and when the bites occurred. For sociodemographic data, participants’ sex, age, education level, and monthly household income in HKD were collected.

### 2.3. Statistical Analysis

Statistical analysis was conducted using IBM SPSS 26. To improve the representativeness of the sample, cases were weighted by age and sex using end-of-2019 census data [24].

Self-rated health and average hours of sleep per day were the dependent variables. Self-rated health was dichotomised into low (≤5) and high (>5) since those who rated ≤5 accounted for about 25% of the weighted sample. Average hours of sleep per day were dichotomised into low (<7 h) and high (≥7 h) since a previous study found that nearly 50% of the Hong Kong population had <7 h of sleep per day [25].

Variables related to bedbug infestation and sociodemographics were the independent variables. Bivariate logistic regression using a chi-square test for categorical variables was used to identify the variables for bedbug infestation associated with dichotomised self-rated health and average hours of sleep per day. Variables related to bedbug infestation were considered for inclusion in multivariable logistic regression if *p*-values were less than or equal to 0.05 in the bivariate analysis; sociodemographic variables were included regardless of their statistical significance.

Multivariable logistic regression was performed to investigate the effect of variables for bedbug infestation on self-rated health and average hours of sleep per day. The unadjusted model was fitted using backward conditional method where covariates related to bedbug infestation were removed stepwise starting from the covariate with the largest *p*-value until all covariates had *p* < 0.05; this model was adjusted by entering the sociodemographic variables (sex, age, education level, monthly household income) regardless of their significance.

The pseudo-R^2^ values for the multivariable regression models were calculated using Nagelkerke’s approach [26]. The Hosmer–Lemeshow goodness-of-fit test was performed on the models, where the goodness-of-fit assumption was not violated if *p* > 0.05. Multicollinearity diagnostics were also performed on the models, where multicollinearity was considered to be present if the covariates had an absolute value of Pearson correlation coefficient |r| ≥ 0.7 or variance inflation factors (VIF) ≥ 10 [27].

Descriptive statistics were presented as mean ± SD or frequencies and percentages. For bivariate logistic regression, effect estimates were presented as odds ratio (OR). For multivariable logistic regression, unadjusted and adjusted OR were presented instead (uOR and aOR). All ORs were presented with their corresponding 95% confidence interval (CI). Statistical significance was considered when the two-sided *p*-value was < 0.05.

## 3. Results

### 3.1. Patterns of Bedbug Bites

The questionnaire received 696 responses; 663 (95.3%) participants remained after listwise deletion of participants with missing age or sex data. Table 1 shows the weighted responses from the survey, where 422 (63.7%) of the participants had had bedbug infestation in the past year. Among them, 227 (53.7%) had been severely to extremely severely troubled by bedbugs in the past year. For impact on daily life in the past month, over 50% reported moderate to severe impact on mental and emotional health (*n* = 223, 52.8%) and sleeping quality (*n* = 239, 56.6%).

Among participants who had had bedbug infestation, 223 (52.9%) experienced ≥five bites in the past month. Around 50% of all bites occurred on the arms (*n* = 202, 47.8%) and legs (*n* = 215, 51%), and the most common reactions to bites were itchiness (*n* = 322, 76.3%), redness and swelling of the skin (*n* = 246, 58.1%), and difficulties sleeping or restlessness (*n* = 125, 29.6%). Over 50% of the bites occurred during sleep (*n* = 230, 54.5%).

### 3.2. Self-Rated Health and Bedbug Infestation

Table 2 shows the results of the bivariate analysis. Increased bedbug infestation and severity of being troubled by bedbugs in the past year were associated with lower self-rated health (*p* < 0.001). Greater impact on daily life in the past month for all reported aspects including physical health, mental and emotional health, sleeping quality, physical appearance, work and academic performance, social activities, avoidance to go home, and spending money on medication or doctor consultation were associated with lower self-rated health (*p* < 0.001). An increased number of bedbug bites in the past month was associated with lower self-rated health (*p* < 0.001). Bites to the legs, arms, whole body, and head and neck were associated with lower self-rated health (*p* < 0.01). Bite reactions that were associated with lower self-rated health were itchiness, redness and swelling of the skin, difficulties sleeping or restlessness, pain at the site of bite, and bleeding at the site of bite (*p* < 0.001). The times of bites that were associated with lower self-rated health were during sleep, irregularly, and watching TV or otherwise being still (*p* < 0.001). Younger age, higher education level, and higher monthly household income were associated with higher self-rated health (*p* < 0.001).

In the final adjusted model with dichotomised self-rated health as the dependent variable (Table 3), physical appearance (*p* = 0.008), spending money on medication or doctor consultation (*p* = 0.04), number of bites in the past month (*p* = 0.023), and irregular time of bites (aOR = 0.42, 95% CI 0.24–0.74, *p* = 0.003) were independently associated with self-rated health. For physical appearance, increased severity of impact on daily life predicts lower self-rated health (aOR < 1, *p* < 0.05). Bedbug infestation became non-significant after adjustment, and in the unadjusted model, having often (uOR = 0.41, 95% CI 0.19–0.89, *p* = 0.025) or very often (uOR = 0.33, 95% CI 0.15–0.76, *p* = 0.009) bedbug infestation were more likely to have lower self-rated health compared with rarely having infestation.

The final adjusted model with dichotomised self-rated health as the dependent variable had a pseudo R-squared of 0.41. The omnibus test of model coefficients was significant (*p* < 0.001); it was better at predicting self-rated health than the null model and was able to correctly predict 79% of self-rated health. The Hosmer–Lemeshow test was not significant (*p* = 0.318); thus, the goodness-of-fit assumption was not violated. The results of multicollinearity diagnostics are provided as a Supplementary Material (Supplementary Table S2). There was no evidence of multicollinearity.

### 3.3. Average Hours of Sleep per Day and Bedbug Infestation

In the bivariate analysis (Table 2), increased bedbug infestation and severity of being troubled by bedbugs in the past year were associated with lower average hours of sleep per day (*p* < 0.01). Greater impact on daily life in the past month for all reported aspects were associated with lower average hours of sleep per day (*p* < 0.01). Increased number of bedbug bites in the past month was associated with lower average hours of sleep per day (*p* = 0.015). Only bites to the whole body were associated with lower average hours of sleep per day (*p* = 0.008). Bite reactions that were associated with lower average hours of sleep per day were itchiness, and difficulties sleeping or restlessness (*p* < 0.05). Only bites during sleep (*p* = 0.031) were associated with lower average hours of sleep per day. Younger age, higher education level, and higher monthly household income were associated with higher average hours of sleep per day (*p* < 0.001).

In the final adjusted model with dichotomised average hours of sleep per day as the dependent variable (Table 4), mental and emotional health (*p* = 0.016) was independently associated with average hours of sleep per day. Having severe (aOR = 0.33, 95% CI 0.15–0.71, *p* = 0.004) mental and emotional health impact on daily life was more likely to have lower average hours of sleep per day compared with having no impact. Difficulties sleeping or restlessness as a physical reaction to bites became non-significant after adjustment, and in the unadjusted model, those with difficulties sleeping or restlessness (uOR = 0.49, 95% CI 0.26–0.89, *p* = 0.02) were more likely to have lower average hours of sleep per day.

The final adjusted model with dichotomised average hours of sleep per day as the dependent variable had a pseudo R-squared of 0.216. The omnibus test of model coefficients was significant (*p* < 0.001), viz, the model was better at predicting average hours of sleep per day than the null model and was able to correctly predict 69.9% of average hours of sleep per day. The Hosmer–Lemeshow test was not significant (*p* = 0.087); thus, the goodness-of-fit assumption was not violated. There was no evidence of multicollinearity (Supplementary Table S2).

## 4. Discussion

This is the first empirical study to investigate the patterns of bedbug bites and their health impact among bedbug victims in Hong Kong, considering bedbugs as a causal agent instead of a potential vector in transmitting diseases. This study found that most (32.7%) participants reported having >10 bites per month; increased number of bites in the past month was independently associated with lower self-rated health. Having many bedbug bites may be explained by a higher level of bedbug infestation and the biting behaviour described as ‘breakfast, lunch, and dinner’ where bedbugs bite multiple sites in a linear pattern during one instance of feeding [28]. Having more bedbug bites can make the related health impact more severe, hence lower self-rated health [9,13,14,29,30,31,32]. Irregular time of bite was also independently associated with lower self-rated health and was the second most common time of bite (37.9%); hence, having bites throughout the day may be more problematic than having bites at specified times of the day such as at night or during sleep.

This study found that bedbug bites most commonly occurred on the arms (47.8%) and legs (51%) which agrees with previous studies [9,33]. With regards to the cutaneous reactions to bedbug bites, the most common were itchiness (76.3%) and redness and swelling of the skin (58.1%), which is similar to a previous cross-sectional study that found inflammation and redness of the skin to occur in 100% of participants who experienced bedbug bites [33]. The difference between the proportion of reported cutaneous reactions between the studies may be explained by the difference in the participants’ sensitivity to bedbug bites [34], or underreporting due to the common difficulty of differentiating between bedbug bites and other arthropod bites or skin conditions [8,30,31,32].

With regards to uncommon or rare reactions to bedbug bites, this study identified one participant who reported blisters which may represent uncommon bullous reactions that are estimated to occur in 6% of bedbug bites [35]. This study also identified some participants who developed systemic reactions to bedbug bites including fever (0.3%), difficulties breathing (0.8%), and headache (3.5%). The rare occurrence of systemic reactions is consistent with the findings of previous bedbug cases and studies [9,29,36,37,38,39]. Aside from bedbug bites, systemic reactions such as headache or nausea may also result from overexposure to pesticides used for treating bedbug infestations [40].

Notwithstanding the apparent inability of bedbugs to transmit vector-borne diseases [4,5,6,7], this study found bedbug infestations to negatively affect various aspects of health, including physical and psychosocial health, which agrees with previous studies [8,9,11,12,13,14,29,30,31,32]. Particularly, physical appearance and spending money on medication or doctor consultation were independently associated with lower self-rated health. Impact on physical appearance from cutaneous reactions to bedbug bites may represent a significant and readily observable adverse effect to health [41]. Spending money on medication or doctor consultation may represent a financial burden to bedbug victims [1,10].

This study found that having bedbug infestations may reduce the quantity and quality of sleep, and the sleep disturbances from bedbug infestation are well reported in previous literature [9,12,29,30,31,32]. This study also found that having severe mental and emotional health impact on daily life from bedbug infestation was independently associated with lower average hours of sleep. Previous studies found several mental health conditions to be associated with bedbug infestations, including those that directly relate to duration of sleep, such as insomnia, or have a more complex relationship with sleep such as depression or PTSD [12,13,14].

### 4.1. Recommendations

Given Hong Kong’s status as an international city involved in global travel and trade, the successful control of bedbugs in Hong Kong may inform the control of the global bedbug resurgence. The same can be said for other international hubs. If bedbug infestation results in adverse health effects in an affluent city such as Hong Kong, we may expect developing countries to experience worse health outcomes from bedbug infestations despite the lack of entomological literature on bedbugs in those countries [4].

Bedbugs are a neglected issue in Hong Kong, and are considered an insignificant pest compared with others that are known vectors for infectious diseases such as mosquitoes [19,23]. Policy evaluation of bedbugs as a threat to public health needs to move beyond considering their ability to transmit vector-borne diseases to bedbugs as an agent (Figure 1), with adverse health effects to bedbug victims that go beyond physical but all aspects of health including mental, social, and financial as demonstrated in this and several other studies [8,9,11,12,13,14,29,30,31,32]. The standard of considering the importance of hematophagous ectoparasites based on their ability to transmit vector-borne diseases, as has been applied to mosquitoes, should not be applied to bedbugs. If scientific and public discourse shifts from considering bedbugs as an incompetent vector to a competent infectious agent in their own right, it may raise public concern to a level that it deserves and motivate efforts and policies against bedbugs.

Public health efforts, laws, and policies to eradicate or alleviate bedbug infestation should address its risk factors, which include crowded housing and poverty [15,21,42,43,44]. Immediate public health interventions such as extermination programs or public education on the affordable self-management strategies for bedbug infestation should be directed to at-risk groups including elderly, lower education level, lower-income households, and those living in at-risk areas such as crowded or dilapidated housing [16,18,45,46,47]. Long-term solutions include addressing housing issues; this may be considered as a ‘best buy’ for addressing bedbug infestations as it can reverberate improvements to other aspects of health and wellbeing including mental health, access to education, employment, etc.; in the context of Hong Kong, this would revolve around alleviating the vulnerabilities of living in subdivided units and providing adequate living space through improved public housing and related policies [21,48,49].

### 4.2. Limitations

The data from this study depend on self-reports by participants, making it difficult to measure bedbug infestation and the effects of their bites. The reductionist survey design made it difficult to comprehensively assess the health condition and sleep quality of the participant. No medical records were available to confirm any health outcomes of bedbug infestation. Furthermore, the survey did not use a standard instrument to assess the subjective sleep quality such as the Pittsburgh Sleep Quality Index (PSQI) or Mini Sleep Questionnaire (MSQ) [50].

There may be a proclivity among bedbug victims to volunteer as participants due to the voluntary online questionnaire being relevant to them, thus the proportion of participants who had had bedbug infestation may be arbitrarily higher.

The responses provided by the participants may be subject to their interpretation. For example, ‘headache’ may be used as a colloquialism by the participants to describe the frustration from troubles brought upon by bedbug infestation rather than the health condition. Although a picture of a bedbug was provided on the survey to minimise erroneous recognition of bedbugs or reporting on the health impact for other arthropod bites, previous study found that older people (>60-year-olds) were more likely to erroneously identify bedbugs from a picture [44], and arthropod bites—from bedbugs or otherwise—may be attributed to the wrong arthropod or other skin conditions [8,30,31,32]. Furthermore, systemic reactions such as headache may be misattributed to bedbug bites instead of from overexposure to pesticides used for treating bedbug infestations [40].

The cross-sectional study design was unable to establish the temporal relationship between the occurrence of bedbug infestation and its health impacts to the participants’ perceived health status and average hours of sleep per day. Low self-rated health or average hours of sleep per day may have existed before the bedbug infestation.

Although the analysis was weighted by age and sex, the use of volunteer sampling and online data collection may lower the representativeness of the sample. The sample may have reduced representativeness for vulnerable or disadvantaged groups who may have limited internet access and be disproportionately affected by bedbug infestation and their health impacts [10,15,16,17,18].

Although the manifestation of bedbug infestation and details of their bites are unlikely to be different across various sociocultural contexts, one should be mindful of the setting in which this study was conducted when generalising the results of this study to other settings. For example, Hong Kong has a subtropical climate, it is a high-income–high-disparity city and is one of most densely populated cities in the world with household crowding, a risk factor of bedbug infestation [15,21,42], to be a major issue.

## 5. Conclusions

Bedbug infestations are a pest of significant health detriment to their victims. This study brings attention to bedbug infestations and their health impacts in Hong Kong by providing empirical evidence describing the patterns of bedbug bites, including the location of bites, when they occurred, and the physical reaction to bites, and how they were associated with lower self-rated health and average hours of sleep per day among the bedbug victims. Bedbug bites impact several dimensions of health including physical, mental, social, and financial. There is a need to consider the threat of bedbugs as agents resulting in adverse health outcomes, beyond their (in)ability to transmit vector-borne diseases. To eradicate bedbugs or alleviate their health burden, public health interventions should be implemented. These may include public education and eradication programs prioritising vulnerable groups such as the elderly or those living in crowded housing, and supportive laws and policies addressing the risk factors to bedbug infestation such as alleviating crowded housing and poverty. The successful control of bedbugs in Hong Kong, an international hub, can inform the control of the global bedbug resurgence.

## Figures and Tables

**Table 1:** Survey responses.

Variable (*n*)	Weighted Freq. (%) or Mean ± SD
Self-rated health (663)	6.66 ± 1.85
Average hours of sleep per day (663)	
<5	132 (19.9%)
5–6	270 (40.8%)
7–9	225 (33.9%)
>9	36 (5.5%)
Bedbug infestation (663)	
Never	241 (36.3%)
Rarely	137 (20.6%)
Sometimes	92 (13.9%)
Often	93 (14.1%)
Very often	100 (15.1%)
Severity of being troubled by bedbugs in the past year (422)	
Not severe at all	19 (4.5%)
Mildly severe	55 (13.1%)
Moderately severe	121 (28.7%)
Severe	124 (29.4%)
Extremely severe	103 (24.3%)
Impact on daily life in the past month	
Physical health (422)	
No impact	143 (33.8%)
Slight	114 (27.0%)
Moderate	109 (25.8%)
Severe	57 (13.5%)
Mental and emotional health (422)	
No impact	117 (27.6%)
Slight	83 (19.6%)
Moderate	114 (27.0%)
Severe	109 (25.8%)
Sleeping quality (422)	
No impact	111 (26.2%)
Slight	73 (17.2%)
Moderate	110 (26.1%)
Severe	128 (30.4%)
Physical appearance (422)	
No impact	167 (39.5%)
Slight	106 (25.0%)
Moderate	99 (23.4%)
Severe	51 (12.1%)
Work and academic performance (422)	
No impact	196 (46.5%)
Slight	107 (25.4%)
Moderate	78 (18.5%)
Severe	40 (9.6%)
Social activities (422)	
No impact	206 (48.7%)
Slight	111 (26.4%)
Moderate	74 (17.5%)
Severe	31 (7.4%)
Avoidance to go home (422)	
No impact	180 (42.5%)
Slight	88 (20.9%)
Moderate	81 (19.3%)
Severe	73 (17.3%)
Spending money on medication or doctor consultation (422)	
No impact	169 (40.0%)
Slight	95 (22.5%)
Moderate	100 (23.6%)
Severe	59 (14.0%)
No. bites in past month (422)	
0	96 (22.7%)
1–4	103 (24.4%)
5–10	85 (20.2%)
>10	138 (32.7%)
Location of bites (422)	
Legs	215 (51.0%)
Arms	202 (47.8%)
Whole body	132 (31.3%)
Head and neck	82 (19.5%)
Chest and back	67 (16.0%)
Belly	49 (11.5%)
Physical reaction to bites (422)	
Itchiness	322 (76.3%)
Redness and swelling of the skin	246 (58.1%)
Difficulties sleeping or restlessness	125 (29.6%)
Pain at the site of bite	82 (19.4%)
Bleeding at the site of bite	36 (8.5%)
Headache	15 (3.5%)
Difficulties breathing	3 (0.8%)
Fever	1 (0.3%)
Blisters	1 (0.3%)
Time of bites (422)	
During sleep	230 (54.5%)
Irregularly	160 (37.9%)
Night (before sleeping)	103 (24.4%)
Watching TV or otherwise being still	76 (18.1%)
Daytime	42 (9.9%)
Holding clothes or umbrellas	13 (3.0%)
Sex (663)	
Female	360 (54.4%)
Male	303 (45.6%)
Age (663)	
0–24	138 (20.8%)
25–44	196 (29.6%)
45–64	210 (31.7%)
≥65	119 (18.0%)
Education level (663)	
Primary education or below	68 (10.3%)
Secondary education	218 (32.8%)
Tertiary education	377 (56.9%)
Monthly household income (663)	
<HKD 10,000	99 (14.9%)
HKD 10,000–30,000	254 (38.3%)
HKD 30,001–50,000	156 (23.5%)
HKD 50,001–80,000	99 (15.0%)
>HKD 80,000	55 (8.4%)

**Table 2:** Bivariate analysis of survey responses by self-rated health and average hours of sleep per day.

Variables	Self-Rated Health (%)		OR (95% CI)	*p*-Value *	Average Hours of Sleep per Day (%)		OR (95% CI)	*p*-Value *
	Low ≤ 5	High > 5			Low < 7	High ≥ 7		
Bedbug infestation								
Never (ref.)	34 (14.1)	207 (85.9)		**<0.001**	115 (47.7)	126 (52.3)		**<0.001**
Rarely	19 (13.9)	118 (86.1)	1.01 (0.55–1.85)	0.975	78 (56.9)	59 (43.1)	0.69 (0.45–1.05)	0.086
Sometimes	17 (18.3)	76 (81.7)	0.73 (0.39–1.40)	0.345	57 (61.3)	36 (38.7)	0.57 (0.35–0.94)	**0.026**
Often	34 (36.6)	59 (63.4)	0.29 (0.16–0.50)	**<0.001**	75 (80.6)	18 (19.4)	0.23 (0.13–0.40)	**<0.001**
Very often	55 (55.0)	45 (45.0)	0.13 (0.08–0.23)	**<0.001**	77 (77.0)	23 (23.0)	0.27 (0.16–0.46)	**<0.001**
Severity of being troubled by bedbugs in the past year								
Not severe at all (ref.)	3 (15.0)	17 (85.0)		**<0.001**	8 (42.1)	11 (57.9)		**0.003**
Mildly severe	4 (7.3)	51 (92.7)	1.78 (0.35–9.19)	0.490	38 (69.1)	17 (30.9)	0.36 (0.12–1.04)	0.058
Moderately severe	19 (15.7)	102 (84.3)	0.82 (0.20–3.33)	0.776	70 (57.9)	51 (42.1)	0.56 (0.21–1.49)	0.245
Severe	43 (34.7)	81 (65.3)	0.29 (0.07–1.13)	0.075	91 (73.4)	33 (26.6)	0.28 (0.11–0.76)	**0.012**
Extremely severe	55 (53.4)	48 (46.6)	0.13 (0.03–0.53)	**0.004**	80 (77.7)	23 (22.3)	0.22 (0.08–0.61)	**0.003**
Impact on daily life in the past month								
Physical health								
No impact (ref.)	18 (12.6)	125 (87.4)		**<0.001**	83 (58.5)	59 (41.5)		**<0.001**
Slight	26 (22.8)	88 (77.2)	0.49 (0.26–0.96)	**0.037**	72 (63.2)	42 (36.8)	0.82 (0.50–1.36)	0.449
Moderate	46 (42.2)	63 (57.8)	0.20 (0.10–0.36)	**<0.001**	84 (77.1)	25 (22.9)	0.41 (0.24–0.72)	**0.002**
Severe	34 (59.6)	23 (40.4)	0.09 (0.05–0.20)	**<0.001**	47 (82.5)	10 (17.5)	0.28 (0.13–0.61)	**0.001**
Mental and emotional health								
No impact (ref.)	13 (11.2)	103 (88.8)		**<0.001**	62 (53.4)	54 (46.6)		**<0.001**
Slight	18 (21.7)	65 (78.3)	0.48 (0.22–1.04)	0.062	49 (59.0)	34 (41.0)	0.81 (0.46–1.43)	0.461
Moderate	40 (35.1)	74 (64.9)	0.24 (0.12–0.47)	**<0.001**	82 (71.9)	32 (28.1)	0.44 (0.26–0.77)	**0.004**
Severe	53 (48.6)	56 (51.4)	0.13 (0.07–0.27)	**<0.001**	94 (86.2)	15 (13.8)	0.18 (0.10–0.35)	**<0.001**
Sleeping quality								
No impact (ref.)	16 (14.5)	94 (85.5)		**<0.001**	67 (60.4)	44 (39.6)		**<0.001**
Slight	10 (13.7)	63 (86.3)	1.10 (0.47–2.58)	0.823	38 (52.1)	35 (47.9)	1.42 (0.78–2.58)	0.251
Moderate	38 (34.5)	72 (65.5)	0.33 (0.17–0.63)	**<0.001**	76 (68.5)	35 (31.5)	0.7 (0.40–1.21)	0.200
Severe	59 (46.1)	69 (53.9)	0.20 (0.11–0.38)	**<0.001**	107 (82.9)	22 (17.1)	0.31 (0.17–0.56)	**<0.001**
Physical appearance								
No impact (ref.)	21 (12.6)	146 (87.4)		**<0.001**	96 (57.5)	71 (42.5)		**<0.001**
Slight	32 (30.2)	74 (69.8)	0.33 (0.18–0.62)	**<0.001**	69 (65.7)	36 (34.3)	0.71 (0.43–1.18)	0.184
Moderate	40 (40.4)	59 (59.6)	0.21 (0.12–0.39)	**<0.001**	75 (75.8)	24 (24.2)	0.44 (0.25–0.76)	**0.003**
Severe	32 (62.7)	19 (37.3)	0.09 (0.04–0.18)	**<0.001**	47 (92.2)	4 (7.8)	0.11 (0.04–0.32)	**<0.001**
Work and academic performance								
No impact (ref.)	40 (20.4)	156 (79.6)		**<0.001**	119 (60.4)	78 (39.6)		**0.004**
Slight	31 (28.7)	77 (71.3)	0.65 (0.38–1.12)	0.118	76 (70.4)	32 (29.6)	0.64 (0.39–1.06)	0.082
Moderate	30 (38.5)	48 (61.5)	0.41 (0.23–0.72)	**0.002**	55 (70.5)	23 (29.5)	0.63 (0.36–1.1)	0.105
Severe	23 (56.1)	18 (43.9)	0.20 (0.10–0.41)	**<0.001**	37 (90.2)	4 (9.8)	0.15 (0.05–0.46)	**<0.001**
Social activities								
No impact (ref.)	34 (16.5)	172 (83.5)		**<0.001**	123 (59.7)	83 (40.3)		**0.002**
Slight	35 (31.3)	77 (68.8)	0.43 (0.25–0.75)	**0.003**	79 (71.2)	32 (28.8)	0.6 (0.37–0.99)	**0.047**
Moderate	34 (45.9)	40 (54.1)	0.23 (0.13–0.42)	**<0.001**	56 (75.7)	18 (24.3)	0.49 (0.27–0.89)	**0.019**
Severe	22 (71.0)	9 (29.0)	0.08 (0.03–0.19)	**<0.001**	29 (93.5)	2 (6.5)	0.12 (0.03–0.47)	**0.002**
Avoidance to go home								
No impact (ref.)	25 (14.0)	154 (86.0)		**<0.001**	101 (56.4)	78 (43.6)		**<0.001**
Slight	22 (24.7)	67 (75.3)	0.51 (0.27–0.96)	**0.038**	64 (72.7)	24 (27.3)	0.49 (0.28–0.85)	**0.011**
Moderate	36 (44.4)	45 (55.6)	0.20 (0.11–0.38)	**<0.001**	62 (76.5)	19 (23.5)	0.4 (0.22–0.72)	**0.002**
Severe	41 (56.2)	32 (43.8)	0.13 (0.07–0.24)	**<0.001**	59 (80.8)	14 (19.2)	0.3 (0.16–0.58)	**<0.001**
Spending money medication or doctor consultation								
No impact (ref.)	30 (17.8)	139 (82.2)		**<0.001**	100 (59.2)	69 (40.8)		**<0.001**
Slight	23 (24.2)	72 (75.8)	0.67 (0.36–1.24)	0.205	61 (64.2)	34 (35.8)	0.81 (0.48–1.37)	0.432
Moderate	33 (33.0)	67 (67.0)	0.44 (0.25–0.78)	**0.005**	71 (71.7)	28 (28.3)	0.58 (0.34–0.99)	**0.047**
Severe	38 (64.4)	21 (35.6)	0.11 (0.06–0.22)	**<0.001**	55 (93.2)	4 (6.8)	0.12 (0.04–0.33)	**<0.001**
No. bites in past month								
0 (ref.)	11 (11.5)	85 (88.5)		**<0.001**	61 (63.5)	35 (36.5)		**0.015**
1–4	22 (21.4)	81 (78.6)	0.48 (0.22–1.06)	0.069	61 (58.7)	43 (41.3)	1.23 (0.70–2.19)	0.471
5–10	18 (21.2)	67 (78.8)	0.49 (0.22–1.11)	0.089	58 (68.2)	27 (31.8)	0.81 (0.44–1.51)	0.515
>10	74 (53.6)	64 (46.4)	0.11 (0.05–0.23)	**<0.001**	107 (77.5)	31 (22.5)	0.51 (0.29–0.91)	**0.022**
Location of bites (no = ref.)								
Legs	81 (37.7)	134 (62.3)	0.44 (0.28–0.67)	**<0.001**	151 (70.2)	64 (29.8)	0.8 (0.53–1.21)	0.291
Arms	74 (36.6)	128 (63.4)	0.50 (0.33–0.77)	**0.002**	144 (71.3)	58 (28.7)	0.74 (0.49–1.12)	0.157
Whole body	54 (40.6)	79 (59.4)	0.47 (0.31–0.74)	**<0.001**	102 (77.3)	30 (22.7)	0.53 (0.33–0.84)	**0.008**
Head and neck	38 (45.8)	45 (54.2)	0.40 (0.24–0.66)	**<0.001**	53 (63.9)	30 (36.1)	1.25 (0.76–2.07)	0.383
Chest and back	21 (31.3)	46 (68.7)	0.89 (0.51–1.57)	0.689	43 (64.2)	24 (35.8)	1.2 (0.70–2.08)	0.507
Belly	11 (22.4)	38 (77.6)	1.48 (0.73–3.00)	0.277	28 (57.1)	21 (42.9)	1.72 (0.94–3.16)	0.080
Physical reaction to bites (no = ref.)								
Itchiness	109 (33.9)	213 (66.1)	0.36 (0.200.65)	**<0.001**	228 (70.8)	94 (29.2)	0.59 (0.37–0.95)	**0.028**
Redness and swelling of the skin	90 (36.6)	156 (63.4)	0.42 (0.26–0.66)	**<0.001**	170 (69.4)	75 (30.6)	0.86 (0.57–1.30)	0.465
Difficulties sleeping or restlessness	58 (46.4)	67 (53.6)	0.33 (0.21–0.52)	**<0.001**	106 (84.1)	20 (15.9)	0.29 (0.17–0.49)	**<0.001**
Pain at the site of bite	39 (48.1)	42 (51.9)	0.36 (0.22–0.59)	**<0.001**	60 (73.2)	22 (26.8)	0.74 (0.43–1.27)	0.277
Bleeding at the site of bite	22 (61.1)	14 (38.9)	0.22 (0.11–0.45)	**<0.001**	27 (77.1)	8 (22.9)	0.62 (0.28–1.38)	0.243
Headache	15 (100)	0 (0)	0 (0–0)	0.998	15 (100)	0 (0)	0 (0–0)	0.998
Difficulties breathing	2 (66.7)	1 (33.3)	0.24 (0.02–2.32)	0.216	2 (66.7)	1 (33.3)	1.22 (0.12–11.96)	0.866
Fever	1 (50.0)	1 (50.0)	0.28 (0.01–9.77)	0.484	1 (100)	0 (0)	0 (0–0)	1
Blisters	0 (0)	1 (100)	676,690,186.31 (0–0)	1	1 (100)	0 (0)	0 (0–0)	1
Time of bites (no = ref.)								
During sleep	84 (36.4)	147 (63.6)	0.47 (0.31–0.73)	**<0.001**	167 (72.6)	63 (27.4)	0.64 (0.42–0.96)	**0.031**
Irregularly	64 (39.8)	97 (60.2)	0.46 (0.30–0.70)	**<0.001**	110 (68.8)	50 (31.3)	0.94 (0.62–1.43)	0.773
Night (before sleeping)	33 (32.0)	70 (68.0)	0.85 (0.52–1.37)	0.500	63 (60.6)	41 (39.4)	1.53 (0.97–2.43)	0.070
Watching TV or otherwise being still	36 (46.8)	41 (53.2)	0.39 (0.24–0.65)	**<0.001**	58 (76.3)	18 (23.7)	0.62 (0.35–1.10)	0.103
Daytime	14 (33.3)	28 (66.7)	0.77 (0.39–1.51)	0.451	29 (69.0)	13 (31.0)	0.95 (0.47–1.88)	0.873
Holding clothes or umbrellas	5 (38.5)	8 (61.5)	0.73 (0.23–2.32)	0.589	8 (61.5)	5 (38.5)	1.16 (0.36–3.73)	0.803
Sex								
Female (ref.)	96 (26.6)	265 (73.4)			226 (62.6)	135 (37.4)		
Male	62 (20.5)	241 (79.5)	1.41 (0.98–2.03)	0.066	176 (58.3)	126 (41.7)	1.19 (0.87–1.63)	0.266
Age								
0–24	21 (15.3)	116 (84.7)	4.06 (2.25–7.31)	**<0.001**	66 (47.8)	72 (52.2)	3.76 (2.18–6.48)	**<0.001**
25–44	43 (22.1)	152 (77.9)	2.60 (1.58–4.27)	**<0.001**	114 (58.2)	82 (41.8)	2.47 (1.48–4.13)	**<0.001**
45–64	43 (20.4)	168 (79.6)	2.92 (1.78–4.80)	**<0.001**	130 (61.9)	80 (38.1)	2.11 (1.27–3.52)	**0.004**
≥65 (ref.)	51 (42.9)	68 (57.1)		**<0.001**	92 (77.3)	27 (22.7)		**<0.001**
Education level								
Primary education or below	44 (64.7)	24 (35.3)	0.12 (0.07–0.21)	**<0.001**	63 (92.6)	5 (7.4)	0.1 (0.04–0.25)	**<0.001**
Secondary education	47 (21.7)	170 (78.3)	0.77 (0.51–1.17)	0.219	136 (62.4)	82 (37.6)	0.71 (0.51–1)	**0.050**
Tertiary education (ref.)	67 (17.7)	311 (82.3)		**<0.001**	204 (54.0)	174 (46.0)		**<0.001**
Monthly household income								
<HKD 10,000	47 (48.0)	51 (52.0)	0.05 (0.01–0.19)	**<0.001**	76 (77.6)	22 (22.4)	0.28 (0.14–0.57)	**<0.001**
HKD 10,000–30,000	72 (28.2)	183 (71.8)	0.12 (0.03–0.44)	**0.001**	164 (64.6)	90 (35.4)	0.53 (0.30–0.95)	**0.034**
HKD 30,001–50,000	28 (18.1)	127 (81.9)	0.21 (0.05–0.79)	**0.022**	81 (51.9)	75 (48.1)	0.89 (0.48–1.64)	0.700
HKD 50,001–80,000	8 (8.1)	91 (91.9)	0.49 (0.11–2.14)	0.345	54 (54.5)	45 (45.5)	0.81 (0.42–1.57)	0.540
>HKD 80,000 (ref.)	2 (3.6)	53 (96.4)		**<0.001**	27 (49.1)	28 (50.9)		**<0.001**

* *p*-values in bold indicate statistically significant results (*p* < 0.05).

**Table 3:** Unadjusted and adjusted logistic regression for dichotomised self-rated health regressed against fitted variables.

Self-Rated Health (High = 1)	uOR (95% CI)	Unadjusted *p*-Value *	aOR (95% CI)	Adjusted *p*-Value *
Bedbug infestation (Rarely = ref.)		**0.018**		0.158
Sometimes	0.94 (0.42–2.07)	0.875	1.14 (0.49–2.68)	0.759
Often	0.41 (0.19–0.89)	**0.025**	0.59 (0.25–1.41)	0.236
Very often	0.33 (0.15–0.76)	**0.009**	0.46 (0.19–1.10)	0.081
Physical appearance (No impact = ref.)		**0.009**		**0.008**
Slight	0.38 (0.17–0.87)	**0.023**	0.33 (0.14–0.81)	**0.015**
Moderate	0.25 (0.11–0.61)	**0.002**	0.21 (0.08–0.54)	**0.001**
Severe	0.20 (0.07–0.56)	**0.002**	0.18 (0.06–0.55)	**0.003**
Spending money medication or doctor consultation (No impact = ref.)		**0.033**		**0.040**
Slight	1.53 (0.69–3.40)	0.293	2.27 (0.93–5.50)	0.070
Moderate	2.18 (0.94–5.06)	0.069	2.42 (0.96–6.13)	0.062
Severe	0.70 (0.27–1.80)	0.456	0.89 (0.31–2.54)	0.824
No. bites in past month (0 = ref.)		**<0.001**		**0.023**
1–4	1.10 (0.43–2.80)	0.850	0.87 (0.32–2.35)	0.783
5–10	2.26 (0.78–6.54)	0.131	2.09 (0.67–6.52)	0.203
>10	0.52 (0.20–1.34)	0.174	0.62 (0.22–1.72)	0.360
Time of bites				
Irregularly	0.39 (0.23–0.66)	**<0.001**	0.42 (0.24–0.74)	**0.003**
Male (Female = ref.)			1.20 (0.69–2.10)	0.513
Age (≥65 = ref.)				0.073
0–24			2.07 (0.66–6.54)	0.214
25–44			0.65 (0.24–1.72)	0.381
45–64			0.80 (0.29–2.20)	0.665
Education level (Tertiary education = ref.)				**0.011**
Primary education or below			0.30 (0.10–0.94)	**0.038**
Secondary education			1.52 (0.83–2.78)	0.175
Monthly household income (>HKD80,000 = ref.)				0.213
<HKD 10,000			0.22 (0.03–1.46)	0.116
HKD 10,000–30,000			0.28 (0.05–1.65)	0.159
HKD 30,001–50,000			0.25 (0.04–1.52)	0.131
HKD 50,001–80,000			0.77 (0.11–5.61)	0.795
Constant	12.50	**<0.001**	33.09	**<0.001**
Pseudo R-squared	0.334		0.410	
Omnibus tests of model coefficients	*p* < 0.001		*p* < 0.001	
% correctly predicted	79.1%		79.0%	
Hosmer–Lemeshow test	0.360		0.318	

* *p*-values in bold indicate statistically significant results (*p* < 0.05).

**Table 4:** Unadjusted and adjusted logistic regression for dichotomised average hours of sleep per day regressed against fitted variables.

Average Hours of Sleep per Day (High = 1)	uOR (95% CI)	Unadjusted *p*-Value *	aOR (95% CI)	Adjusted *p*-Value *
Mental and emotional health (No impact = ref.)		**0.003**		**0.016**
Slight	0.87 (0.49–1.54)	0.635	1 (0.54–1.84)	0.993
Moderate	0.58 (0.32–1.04)	0.068	0.60 (0.32–1.11)	0.104
Severe	0.27 (0.13–0.55)	**<0.001**	0.33 (0.15–0.71)	**0.004**
Physical reaction to bites				
Difficulties sleeping or restlessness	0.49 (0.26–0.89)	**0.020**	0.64 (0.34–1.23)	0.179
Male (Female = ref.)			1.04 (0.66–1.64)	0.860
Age (≥65 = ref.)				**0.005**
0–24			5.17 (1.76–15.26)	**0.003**
25–44			2.50 (0.90–6.92)	0.079
45–64			1.94 (0.70–5.38)	0.201
Education level (Tertiary education = ref.)				0.333
Primary education or below			0.41 (0.12–1.41)	0.157
Secondary education			1.04 (0.62–1.74)	0.890
Monthly household income (>HKD80,000 = ref.)				0.242
<HKD 10,000			0.55 (0.18–1.70)	0.298
HKD 10,000–30,000			0.34 (0.13–0.89)	**0.029**
HKD 30,001–50,000			0.47 (0.18–1.26)	0.133
HKD 50,001–80,000			0.50 (0.18–1.37)	0.178
Constant	0.88	0.491	0.78	0.692
Pseudo R-squared	0.125		0.216	
Omnibus tests of model coefficients	*p* < 0.001		*p* < 0.001	
% correctly predicted	67.9%		69.9%	
Hosmer–Lemeshow test	0.623		0.087	

* *p*-values in bold indicate statistically significant results (*p* < 0.05).

## Data Availability

The data presented in this study are available as a Supplementary Material (Supplementary Table S3).

## Supplementary Materials

The following are available online at https://www.mdpi.com/article/10.3390/insects12111027/s1, Supplementary Table S1: English survey, Supplementary Table S2: Multicollinearity diagnostics results, Supplementary Table S3: Data.
