# Supplementary material for: The Impact of Bedbug (Cimex spp.) Bites on Self-Rated Health and Average Hours of Sleep per Day: A Cross-Sectional Study among Hong Kong Bedbug Victims
Source: Insects. 2021 Nov 14;12(11):1027. doi: 10.3390/insects12111027 (PMC8623092; doi:10.3390/insects12111027)
Supplement: Supplementary file 1 [file insects-12-01027-s001.zip › English survey.pdf]

The Chinese University of Hong Kong

Questionnaire on Bedbug Infestation Control in Hong Kong

This is a research survey on bedbug infestations in Hong Kong. It aims to understand the bedbug situation in Hong Kong, its impact, and how the housing situation is related to infestations. Results from this study are expected to help residents deal with bedbug infestations in the long run. You are eligible to participate if you are at least 18-year-old and living in Hong Kong.

This research has been approved by the Survey and Behavioural Research Ethics Committee of The Chinese University of Hong Kong.

By agreeing to participate in this research, you allow the research team to collect your survey responses. If follow-up visits are arranged, you agree to allow the research team to take pictures of your home to better understand the bedbug infestation. The information you provide to us will be kept strictly confidential and will only be used for this research. It takes about 20 minutes to complete this questionnaire.

Participating in this research will not bring you any personal benefits. Your participation is completely voluntary. You may refuse to participate in the research and withdraw from the questionnaire even while it is in progress. Your decision to refuse or withdraw from the study will not lead to any adverse consequences.

If you have any questions about this research now or in the future, please contact researcher Wong Hung (Tel: 3943 7510 Email: hwong@cuhk.edu.hk). If you want to know more about your rights as a research participant, please contact the Survey and Behavioural Research Ethics Committee of The Chinese University of Hong Kong (email: fssc02@cuhk.edu.hk).

If you understand the above information and are willing to participate in this research, please select "Agree".

☐Agree

---

1. Please rate your health status today on a scale of 1 (worse possible health you can imagine) to 10 (best possible health you can imagine) below.

1 2 3 4 5 6 7 8 9 10

---

2. Over the past month, how many hours of sleep do you have each day?

☐<5 hours
☐5-6 hours
☐7-9 hours
☐>9 hours

---

3. In the past year, how often did you see bedbugs in your place of residence?

☐Very often
☐Often
☐Sometimes
☐Rarely
☐Never (skip to question 10)

---

4. In the past year, have you been severely troubled by bedbugs?

☐Not severe at all
☐Mildly severe
☐Moderately severe
☐Severe

☐Extremely severe

5. In the past month, how severely has bedbugs impacted your daily life?

	No impact	Slight	Moderate	Severe
Physical health	☐	☐	☐	☐
Mental and emotional health	☐	☐	☐	☐
Sleeping quality	☐	☐	☐	☐
Physical appearance	☐	☐	☐	☐
Work and academic performance	☐	☐	☐	☐
Social activities	☐	☐	☐	☐
Avoidance to go home	☐	☐	☐	☐
Spend money buying medication or seeing the doctor	☐	☐	☐	☐

6. In the past month, how many times have you been bitten by bedbugs?
☐0
☐1-4
☐5-10
☐>10

7. Where did bedbugs bite you? (Multiple selection allowed, leave blank if none)
☐Whole body
☐Head and neck
☐Chest and back
☐Belly
☐Arms
☐Legs
☐Others (please specify): ___________

8. What was the reaction after being bitten by bedbugs? (Multiple selection allowed, leave blank if none)
☐Redness and swelling of the skin
☐Itchiness
☐Pain at the site of bite
☐Bleeding at the site of bite
☐Fever
☐Difficulties breathing
☐Headache
☐Difficulties sleeping or restlessness
☐Others (please specify): ___________

9. When do bedbugs bite you? (Multiple selection allowed)
☐Daytime
☐Night (before sleeping)
☐During sleep
☐Watching TV or otherwise being still
☐Holding clothes or umbrellas
☐Irregularly
☐Other times (please specify): ___________

10. Sex
☐Male
☐Female

11. Age

☐0-24
☐25-44
☐45-64
☐≥65

12. Education level
☐Primary education or below
☐Secondary education
☐Tertiary education

13. Monthly household income (HKD)
☐<HKD10,000
☐HKD10,000-30,000
☐HKD30,001-50,000
☐HKD50,001-80,000

Please leave your contact information if you would like to be involved in future bedbug related research conducted by the CUHK Anti-bedbug Research Action Group.
Telephone: __________
Email: __________
