# Supplementary material for: The Impact of Bedbug (Cimex spp.) Bites on Self-Rated Health and Average Hours of Sleep per Day: A Cross-Sectional Study among Hong Kong Bedbug Victims
Source: Insects. 2021 Nov 14;12(11):1027. doi: 10.3390/insects12111027 (PMC8623092; doi:10.3390/insects12111027)
Supplement: Supplementary file 1 [file insects-12-01027-s001.zip › Multicollinearity diagnostics results.pdf]

Multicollinearity diagnostics for adjusted logistic regression for dichotomised self-rated health regressed against fitted variables.

VIF, r		Bedbug infestation			Physical appearance			Spending money on medication or doctor consultation			No. bites in past month			Time of bites		Age			Education level		Monthly household income			
		Sometimes	Often	Very often	Slight	Moderate	Severe	Slight	Moderate	Severe	1-4	5-10	>10	Irregularly	Female	0-24	25-44	45-64	Primary education or below	Secondary education	<HKD10,000	HKD10,000-30,000	HKD30,001-50,000	HKD50,001-80,000
Bedbug infestation	Sometimes		1.2, -0.163***	1.24, -0.169***	1.58, 0.115*	1.58, 0.013	1.58, -0.128**	1.57, 0.12*	1.53, 0.084	1.58, -0.165***	1.57, 0.109*	1.58, 0.027	1.57, -0.103*	1.57, 0.021	1.57, -0.065	1.57, -0.009	1.57, 0.096*	1.54, -0.068	1.58, 0.013	1.58, 0.035	1.58, -0.05	1.58, 0.035	1.58, -0.004	1.58, -0.016
	Often	1.51, -0.163***		1.33, -0.17***	1.99, -0.026	1.98, 0.117*	1.99, 0.066	1.97, 0.069	1.9, 0.078	1.92, 0.086	1.97, -0.116*	1.92, 0.229***	1.93, 0.043	1.95, 0.038	1.98, -0.023	1.93, -0.037	1.91, -0.007	1.86, -0.146***	1.96, 0.14***	1.99, 0.001	1.95, 0.188***	1.96, 0.037	1.97, -0.062	1.99, -0.085*
	Very often	1.9, -0.169***	1.62, -0.17***		2.42, -0.023	2.41, 0.134**	2.4, 0.296***	2.42, -0.183***	2.33, 0.169***	2.31, 0.357***	2.35, -0.107*	2.36, -0.086	2.18, 0.426***	2.39, 0.026	2.42, 0.046	2.38, -0.112**	2.38, -0.032	2.38, 0.004	2.42, 0.244***	2.42, 0.045	2.41, 0.094*	2.4, 0.173***	2.41, -0.078*	2.42, -0.149***
Physical appearance	Slight	2, 0.115*	2.01, -0.026	2.01, -0.023		1.31, -0.319***	1.52, -0.214***	1.82, 0.3***	1.83, -0.01	1.82, -0.008	1.94, 0.114*	1.89, 0.072	1.89, 0.058	2, 0.132**	1.96, 0.119*	1.99, 0.03	1.99, 0.05	1.99, -0.032	2.01, -0.017	2, -0.033	1.98, -0.018	1.98, -0.009	1.97, 0.123*	2, -0.012
	Moderate	2.22, 0.013	2.22, 0.117*	2.22, 0.134**	1.46, -0.319***		1.61, -0.205***	2.12, -0.049	1.8, 0.415***	2.01, -0.014	2.19, -0.067	2.11, 0.09	2.1, 0.188***	2.23, 0.064	2.21, 0.056	2.19, -0.004	2.2, 0.049	2.21, -0.063	2.23, 0.011	2.22, -0.014	2.2, 0.103*	2.22, 0.028	2.23, -0.062	2.23, -0.06
	Severe	2.1, -0.128**	2.1, 0.066	2.08, 0.296***	1.59, -0.214***	1.52, -0.205***		2.04, -0.118*	1.95, -0.027	1.63, 0.502***	2.08, -0.114*	2.03, 0.05	2.02, 0.233***	2.1, 0.034	2.1, -0.11*	2.08, -0.021	2.09, -0.011	2.1, -0.103*	2.1, 0.172***	2.1, -0.015	2.09, 0.051	2.09, 0.096*	2.09, -0.049	2.1, -0.084
Spending money on medication or doctor consultation	Slight	1.86, 0.12*	1.86, 0.069	1.87, -0.183***	1.7, 0.3***	1.78, -0.049	1.82, -0.118*		1.34, -0.299***	1.5, -0.217***	1.7, 0.193***	1.75, 0.073	1.75, 0.009	1.87, 0.094	1.87, 0.001	1.87, -0.022	1.88, 0.069	1.87, -0.041	1.87, 0.015	1.88, -0.017	1.88, 0.031	1.88, -0.04	1.87, 0.043	1.87, 0.046
	Moderate	2.17, 0.084	2.15, 0.078	2.16, 0.169***	2.05, -0.01	1.82, 0.415***	2.09, -0.027	1.61, -0.299***		1.59, -0.224***	2.22, -0.069	2.21, 0.091	2.2, 0.168***	2.21, 0.147**	2.25, 0.037	2.24, -0.086	2.25, 0.082	2.25, -0.035	2.25, -0.013	2.25, -0.002	2.24, 0.052	2.24, 0.02	2.25, -0.016	2.25, -0.072
	Severe	2.3, -0.165***	2.23, 0.086	2.2, 0.357***	2.09, -0.008	2.09, -0.014	1.79, 0.502***	1.85, -0.217***	1.63, -0.224***		2.29, -0.115*	2.28, 0.051	2.28, 0.249***	2.31, 0.024	2.29, -0.103*	2.3, -0.054	2.31, -0.072	2.31, -0.055	2.29, 0.233***	2.31, -0.02	2.3, 0.057	2.31, 0.098*	2.31, -0.046	2.31, -0.092
No. bites in past month	1-4	2.16, 0.109*	2.16, -0.116*	2.11, -0.107*	2.1, 0.114*	2.13, -0.067	2.15, -0.114*	1.98, 0.193***	2.15, -0.069	2.16, -0.115*		1.36, -0.286***	1.28, -0.397***	2.15, 0.02	2.17, -0.061	2.17, 0.072	2.18, 0.044	2.18, 0.1*	2.17, -0.229***	2.18, 0.033	2.17, -0.106*	2.17, 0	2.18, 0.049	2.17, 0.089
	5-10	2.5, 0.027	2.42, 0.229***	2.44, -0.086	2.36, 0.072	2.38, 0.09	2.42, 0.05	2.34, 0.073	2.46, 0.091	2.48, 0.051	1.57, -0.286***		1.33, -0.35***	2.45, 0.136**	2.51, -0.032	2.49, -0.006	2.48, 0.027	2.42, -0.155**	2.5, -0.04	2.51, 0.024	2.49, -0.053	2.51, 0.075	2.5, -0.01	2.5, -0.074
	>10	3.39, -0.103*	3.31, 0.043	3.07, 0.426***	3.21, 0.058	3.21, 0.188***	3.28, 0.233***	3.18, 0.009	3.34, 0.168***	3.37, 0.249***	2, -0.397***	1.81, -0.35***		3.36, 0.09	3.41, 0.059	3.41, -0.071	3.4, 0.023	3.41, -0.151**	3.36, 0.353***	3.41, -0.065	3.41, 0.232***	3.4, 0.046	3.41, -0.074	3.41, -0.121*
Time of bites	Irregularly	1.18, 0.021	1.16, 0.038	1.17, 0.026	1.18, 0.132**	1.18, 0.064	1.18, 0.034	1.18, 0.094	1.17, 0.147**	1.18, 0.024	1.17, 0.02	1.16, 0.136**	1.17, 0.09		1.16, -0.122*	1.18, -0.002	1.17, -0.047	1.17, -0.097*	1.18, 0.098*	1.18, 0.022	1.17, 0.133**	1.17, 0.056	1.18, -0.041	1.18, -0.103*
Female		1.12, -0.065	1.13, -0.023	1.13, 0.046	1.1, 0.119*	1.12, 0.056	1.13, -0.11*	1.13, 0.001	1.13, 0.037	1.12, -0.103*	1.13, -0.061	1.13, -0.032	1.13, 0.059	1.11, -0.122*		1.13, 0.054	1.13, -0.055	1.13, -0.002	1.12, -0.013	1.13, -0.073	1.12, 0.035	1.13, -0.017	1.13, -0.03	1.13, 0.044
Age	0-24	2.9, -0.009	2.83, -0.037	2.87, -0.112**	2.89, 0.03	2.87, -0.004	2.9, -0.021	2.91, -0.022	2.9, -0.086	2.91, -0.054	2.91, 0.072	2.89, -0.006	2.92, -0.071	2.91, -0.002	2.92, 0.054		1.33, -0.331***	1.36, -0.349***	2.25, -0.158***	2.87, -0.114**	2.9, -0.115**	2.92, 0.043	2.88, 0.096*	2.91, -0.03
	25-44	3.56, 0.096*	3.45, -0.007	3.52, -0.032	3.55, 0.05	3.54, 0.049	3.57, -0.011	3.58, 0.069	3.58, 0.082	3.58, -0.072	3.58, 0.044	3.55, 0.027	3.57, 0.023	3.53, -0.047	3.57, -0.055	1.64, -0.331***		1.32, -0.441***	2.65, -0.202***	3.58, 0.039	3.53, -0.157***	3.58, 0.037	3.57, 0.062	3.58, 0.074
	45-64	3.73, -0.068	3.6, -0.146***	3.77, 0.004	3.81, -0.032	3.8, -0.063	3.83, -0.103*	3.83, -0.041	3.83, -0.035	3.84, -0.055	3.84, 0.1*	3.71, -0.155**	3.83, -0.151**	3.8, -0.097*	3.83, -0.002	1.78, -0.349***	1.41, -0.441***		3.02, -0.173***	3.83, 0.108**	3.72, -0.164***	3.83, -0.017	3.84, 0	3.84, 0.116**
Education level	Primary	2.73, 0.013	2.7, 0.14***	2.73, 0.244***	2.73, -0.017	2.73, 0.011	2.73, 0.172***	2.72, 0.015	2.73, -0.013	2.7, 0.233***	2.72, -0.229***	2.72, -0.04	2.69, 0.353***	2.72, 0.098*	2.7, -0.013	2.1, -0.158***	2.02, -0.202***	2.15, -0.173***		2.48, -0.236***	2.66, 0.455***	2.73, -0.057	2.72, -0.187***	2.72, -0.142***

	**education or below**																							
	**Secondary education**	1.33, 0.035	1.34, 0.001	1.33, 0.045	1.33, -0.033	1.33, -0.014	1.33, -0.015	1.34, -0.017	1.34, -0.002	1.34, -0.02	1.33, 0.033	1.34, 0.024	1.34, -0.065	1.34, 0.022	1.33, -0.073	1.31, -0.114**	1.34, 0.039	1.33, 0.108**	1.21, -0.236***		1.25, 0.032	1.25, 0.224***	1.3, -0.075	1.32, -0.132***
**Monthly household income**	**<HKD10,000**	4.22, -0.05	4.15, 0.188***	4.22, 0.094*	4.17, -0.018	4.18, 0.103*	4.2, 0.051	4.23, 0.031	4.22, 0.052	4.22, 0.057	4.22, -0.106*	4.2, -0.053	4.22, 0.232***	4.17, 0.133**	4.17, 0.035	4.2, -0.115**	4.17, -0.157***	4.1, -0.164***	4.12, 0.455***	3.97, 0.032		1.7, -0.33***	2.27, -0.231***	2.59, -0.175***
	**HKD10,000-30,000**	5.43, 0.035	5.37, 0.037	5.38, 0.173***	5.37, -0.009	5.41, 0.028	5.41, 0.096*	5.43, -0.04	5.42, 0.02	5.43, 0.098*	5.42, 0	5.43, 0.075	5.41, 0.046	5.39, 0.056	5.42, -0.017	5.43, 0.043	5.43, 0.037	5.42, -0.017	5.43, -0.057	5.07, 0.224***	2.18, -0.33***		1.73, -0.437***	2.36, -0.331***
	**HKD30,001-50,000**	3.8, -0.004	3.76, -0.062	3.77, -0.078*	3.73, 0.123*	3.79, -0.062	3.78, -0.049	3.79, 0.043	3.79, -0.016	3.8, -0.046	3.8, 0.049	3.79, -0.01	3.79, -0.074	3.78, -0.041	3.79, -0.03	3.75, 0.096*	3.79, 0.062	3.8, 0	3.79, -0.187***	3.7, -0.075	2.03, -0.231***	1.21, -0.437***		1.82, -0.232***
	**HKD50,001-80,000**	2.77, -0.016	2.78, -0.085*	2.78, -0.149***	2.76, -0.012	2.78, -0.06	2.78, -0.084	2.77, 0.046	2.78, -0.072	2.78, -0.092	2.78, 0.089	2.78, -0.074	2.78, -0.121*	2.78, -0.103*	2.77, 0.044	2.78, -0.03	2.78, 0.074	2.78, 0.116**	2.77, -0.142***	2.76, -0.132***	1.7, -0.175***	1.21, -0.331***	1.33, -0.232***	

Note: ***p<0.001; **p<0.01; *p>0.05

Multicollinearity diagnostics for adjusted logistic regression for dichotomised average hours of sleep per day regressed against fitted variables.

VIF, r		Mental and emotional health			Physical reaction to bites		Age			Education level		Monthly household income			
		Slight	Moderate	Severe	Difficulties sleeping or restlessness	Female	0-24	25-44	45-64	Primary education or below	Secondary education	<HKD10,000	HKD10,000-30,000	HKD30,001-50,000	HKD50,001-80,000
**Mental and emotional health**	**Slight**		1.17, -0.3***	1.14, -0.291***	1.44, -0.185***	1.45, -0.057	1.45, -0.008	1.45, 0.06	1.45, 0.041	1.44, -0.093	1.45, 0.134**	1.45, -0.146**	1.43, 0.076	1.43, 0.06	1.45, -0.011
	**Moderate**	1.4, -0.3***		1.2, -0.358***	1.57, 0.166***	1.75, 0.044	1.74, 0.025	1.73, -0.048	1.74, -0.017	1.74, 0.13**	1.74, -0.159**	1.71, 0.226***	1.72, -0.204***	1.72, 0.009	1.72, 0.069
	**Severe**	1.62, -0.291***	1.42, -0.358***		1.7, 0.391***	2.06, -0.016	2.05, -0.072	2.06, 0.057	2.04, -0.161***	2.04, 0.136**	2.06, -0.011	2.05, -0.005	1.98, 0.237***	2, -0.066	2.04, -0.168***
**Physical reaction to bites**	**Difficulties sleeping or restlessness**	1.49, -0.185***	1.36, 0.166***	1.25, 0.391***		1.51, -0.025	1.5, -0.067	1.49, -0.107*	1.51, -0.11*	1.51, 0.299***	1.5, -0.123*	1.49, 0.242***	1.5, 0.088	1.51, -0.108*	1.51, -0.193***
	**Female**	1.04, -0.057	1.04, 0.044	1.04, -0.016	1.04, -0.025		1.04, 0.054	1.04, -0.055	1.04, -0.002	1.03, -0.013	1.03, -0.073	1.02, 0.035	1.03, -0.017	1.04, -0.03	1.04, 0.044
**Age**	**0-24**	2.7, -0.008	2.69, 0.025	2.68, -0.072	2.68, -0.067	2.69, 0.054		1.3, -0.331***	1.3, -0.349***	2.07, -0.158***	2.64, -0.114**	2.68, -0.115**	2.69, 0.043	2.65, 0.096*	2.69, -0.03
	**25-44**	3.24, 0.06	3.23, -0.048	3.25, 0.057	3.21, -0.107*	3.25, -0.055	1.56, -0.331***		1.27, -0.441***	2.42, -0.202***	3.25, 0.039	3.2, -0.157***	3.25, 0.037	3.23, 0.062	3.24, 0.074
	**45-64**	3.32, 0.041	3.32, -0.017	3.29, -0.161***	3.31, -0.11*	3.32, -0.002	1.6, -0.349***	1.3, -0.441***		2.62, -0.173***	3.32, 0.108**	3.2, -0.164***	3.31, -0.017	3.32, 0	3.32, 0.116**
**Education level**	**Primary education or below**	2.41, -0.093	2.41, 0.13**	2.41, 0.136**	2.42, 0.299***	2.41, -0.013	1.87, -0.158***	1.81, -0.202***	1.92, -0.173***		2.21, -0.236***	2.35, 0.455***	2.43, -0.057	2.42, -0.187***	2.42, -0.142***
	**Secondary education**	1.35, 0.134**	1.35, -0.159**	1.36, -0.011	1.35, -0.123*	1.35, -0.073	1.33, -0.114**	1.36, 0.039	1.36, 0.108**	1.24, -0.236***		1.26, 0.032	1.26, 0.224***	1.32, -0.075	1.34, -0.132***
**Monthly household income**	**<HKD10,000**	3.96, -0.146**	3.87, 0.226***	3.94, -0.005	3.91, 0.242***	3.89, 0.035	3.93, -0.115**	3.9, -0.157***	3.82, -0.164***	3.84, 0.455***	3.68, 0.032		1.71, -0.33***	2.21, -0.231***	2.47, -0.175***
	**HKD10,000-30,000**	5.15, 0.076	5.16, -0.204***	5.02, 0.237***	5.19, 0.088	5.2, -0.017	5.21, 0.043	5.23, 0.037	5.21, -0.017	5.22, -0.057	4.84, 0.224***	2.26, -0.33***		1.72, -0.437***	2.34, -0.331***
	**HKD30,001-50,000**	3.58, 0.06	3.57, 0.009	3.53, -0.066	3.62, -0.108*	3.62, -0.03	3.57, 0.096*	3.61, 0.062	3.62, 0	3.62, -0.187***	3.53, -0.075	2.02, -0.231***	1.2, -0.437***		1.77, -0.232***
	**HKD50,001-80,000**	2.69, -0.011	2.66, 0.069	2.67, -0.168***	2.7, -0.193***	2.69, 0.044	2.69, -0.03	2.69, 0.074	2.7, 0.116**	2.69, -0.142***	2.68, -0.132***	1.69, -0.175***	1.21, -0.331***	1.32, -0.232***	

Note: ***p<0.001; **p<0.01; *p>0.05
